# Daphnane diterpenes inhibit the metastatic potential of B16F10 murine melanoma cells in vitro and in vivo

**DOI:** 10.1186/s12885-018-4693-y

**Published:** 2018-08-29

**Authors:** Myra O. Villareal, Yuki Sato, Kyoko Matsuyama, Hiroko Isoda

**Affiliations:** 1 Faculty of Life and Environmental Sciences, University of Tsukuba, Tsukuba City, 305-8572 Japan; 2 Alliance for Research on North Africa (ARENA), University of Tsukuba, Tsukuba City, 305-8572 Japan; 3 Graduate School of Life and Environmental Sciences, University of Tsukuba, Tsukuba City, 305-8572 Japan

**Keywords:** Melanoma, MMPs, *Id2*, *Mitf*, Daphnane diterpenes, Gnidilatidin

## Abstract

**Background:**

Melanoma is one of the most invasive and aggressive types of cancer with a very poor prognosis. Surgery remains the most efficient treatment prior melanoma invasion and metastasis formation. However, therapy becomes a challenge once the cancer cells colonized other tissues. At present, there are two main classes of therapies acting with a certain efficiency on metastatic melanoma: immune check point inhibitors (anti-PD1/PDL1) and targeted therapy such as Vemurafenib. Unfortunately, these therapies are not fully responsive, induce resistance and/or generate unwanted side effects. In this respect, it is important to continue to discover new cancer therapeutics. Here, we show that daphnane diterpenes type of compounds can prevent melanoma metastasis by inhibiting metastasis-associated matrix metalloproteinases expression without cytotoxicity.

**Methods:**

Evaluation of the anti-metastasis effect of daphnane diterpenes-rich *Thymelaea hirsuta* extract (TH) and its bioactive component gnidilatidin was carried out in vitro using B16 murine melanoma cells and in vivo using male C57BL/6 J mice. Global gene expression in B16 cells was done using DNA microarray, validated using real-time PCR, to further understand the effect of daphnane diterpenes, specifically daphnane diterpenoid gnidilatidin.

**Results:**

Oral administration of daphnane diterpenes-rich *Thymelaea hirsuta* extract (TH) suppressed MMP2 and MMP9 expression, decreasing lung tumor in mice injected with B16 murine melanoma cells. Validation of these observations in vitro showed reduced B16 cells migration, adhesion, and invasion. Results of microarray analysis of B16 cells treated with daphnane diterpenoid gnidilatidin from TH revealed an upregulation of tumor suppressor *Egr1* while inhibiting metastasis-associated genes *Id2* and *Sytl2* expression. A downregulation of the melanoma oncogene microphthalmia-associated transcription factor (*Mitf*) was observed, and most likely caused by the inhibition of *Id2*, a gene that regulated HLH transcription factors such as MITF and also reported to promote tumor cell migration and invasion.

**Conclusions:**

Daphnane diterpenes have inhibitory effect on the metastatic potential of B16 melanoma cells, and the results of this study provided evidence for their potential for use in the prevention and inhibition of melanoma metastasis.

**Electronic supplementary material:**

The online version of this article (10.1186/s12885-018-4693-y) contains supplementary material, which is available to authorized users.

## Background

Melanoma is the most aggressive type of cancer, and among solid tumors, its incidence is rising faster, with a global incidence of about 48,000 and 160,000 deaths annually [1]. Surgery remains an attractive option for treatment of melanoma coupled with chemotherapy that requires long-term treatment that often do not achieve the expected response nor provide evidence for their efficacy [2]. Moreover, therapy becomes a challenge once the cancer cells metastasize and colonize other tissues. Despite significant leaps in terms of diagnosis, surgery, and patient care, highly invasive and metastatic melanoma, that has disseminated to distant sites and the visceral organs and the brain, is almost always incurable, decreasing the medial survival time to only 6–9 months [2–4], making it one of the most therapeutically challenging diseases [5]. Curative treatments for patients with metastatic melanoma remain elusive while available treatments have severe adverse effects. Ipilimumab, for example, is associated with immune-mediated diarrhea and colitis; dacarbazine with respiratory toxicity and dyspnea [6]. Cell-matrix adhesion molecules, integrins, and their interaction with extracellular matrix promotes cell movement or metastasis [7] while matrix metalloproteinases (MMPs) degrade the matrix proteins and regulate cell activities with relevance for cancer, playing a crucial role in the cell migration and metastasis formation [8]. Among the MMPs, MMP9 form a complex with CD44, a cell surface molecule that mediates cell migration and resistance to anti-cancer drugs [9, 10]. So far, studies on the use of small molecule inhibitors for MMPs (for a variety of tumor types) that have reached clinical trials, have been unsuccessful [11]. Naturally occurring daphnane-type diterpene orthoesters have antileukemia, anticancer, toxic, or neurotrophic effects [12] and therefore provide an alternative source of cancer therapeutics. We have previously reported on the melanogenesis inhibitory effects of daphnane diterpenes-rich *Thymealea hirsuta* [13] and two of its daphnane diterpenoids components, hirsein A and hirsein B [14]. Hirseins A and B downregulated microphthalmia-associated transcription factor (MITF) leading to melanogenesis inhibition. In melanoma cells, reduction of the MITF activity has been observed to sensitize the cancer cells to chemotherapeutic agents [15]. MITF also controls melanoma proliferation and invasiveness via regulation of Dia1 [16]. Daphnane diterpenoid mezerein in combination with recombinant human fibroblast interferon (IFN-beta) has antiproliferative properties in human melanoma cells and functions as a negative regulator of melanoma progression [17]. *T. hirsuta* extract contains at least six daphnane diterpenes: hirseins A and B [18], gnidicin, gniditrin, genkwadaphnin, and gnidilatidin [19]. Here, we investigated the effects of daphnane diterpenes-rich *T. hirsuta* extract on the metastatic potential of B16F10 cells in vivo*,* using syngeneic male C57BL/6 J mice, and in vitro using the B16F10 melanoma cells known to be malignant melanoma cells that are stable in their metastatic potential. Since TH contains not just daphnane diterpenes, the possible molecular mechanism underlying the effect of TH was determined in vitro using one of TH components - daphnane diterpene gnidilatidin.

## Methods

### Animals/declarations for the animal research

Six (per treatment group) seven (7)-weeks-old male C57BL/6 J mice (Charles River Laboratories Japan, Inc.) were housed individually in polycarbonate cage lined with paper bedding (Palsoft Oriental Yeast Co., Ltd., Tokyo, Japan) with stainless wire cover and maintained under standard conditions with free access to food and water, and housed in a 12-h light/dark cycle room. The animals were sacrificed using the cervical spine dislocation method. All the experiments complied with the guidelines of the University of Tsukuba’s Regulation of Animal Experiments and were approved by the University of Tsukuba’s Committee on Animal Care and Use (No. 16–046).

### Cell culture

B16F10 murine melanoma cells (B16 cells) were obtained from RIKEN, Tsukuba (Catalog No. RCB2630:B16F10) and cultured in RPMI1640 (Gibco, Invitrogen GmbH, Karlsruhe, Germany) supplemented with 10% fetal bovine serum (FBS) and incubated at 37 °C in a humidified atmosphere of 5% CO_2_. For mouse tail vein injection, 1 × 10^6^ cells/ml B16 cells were resuspended in saline solution, which was then passed through a 79-μm-cell strainer (BD Falcon, BD Biosciences, San Jose, CA, USA) before injection to remove aggregated cells.

### Samples

Daphnane diterpenes were treated as ethanolic extract of air-dried *Thymelaea hirsuta* leaves (10 g in 100 mL of 70% EtOH). *T. hirsuta* extract (TH) was passed through a 0.22 μm filter (Millipore, Germany) and stored at − 80 °C °C until use. Ethanol in the TH samples for oral administration was removed by evaporation (SCRUM Inc., Tokyo, Japan) and dissolved in distilled water. Gnidilatidin (MW: 648.749 g/mol) was extracted from *T. hirsuta* following a similar protocol for isolating hirsein A and hirsein B, as previously reported [18] TH (100 g/l 70% EtOH) contains about 0.5 mg gnidilatidin. Preliminary screening assays using different concentrations of gnidilatidin (data not shown) revealed that gnidilatidin was effective but not cytotoxic at concentrations of 0.1 to 1.0 μM so for this study, gnidilatidin was used at 0.1 μM concentration in all the experiments.

### Tumorigenesis assays

C57BL/6 J mice (7 mice/group) were allowed to acclimatize for a week before they were randomly divided into four groups: Controls of “(−) B16F10” group injected with water and given tap water and “(+)B16F10 group” injected with B16F10 cells and given tap water; “DTIC/(+)B16F10 group” injected with B16F10 cells and orally-administered with 70 mg/kg/day dacarbazine (DTIC) and is the positive control; the “TH*/*(+)B16F10 group” injected with B16F10 cells and orally-administered with 50 mg/kg/day *T. hirsuta* extract. After acclimatization, mice lateral tail veins were injected with B16F10 cells (2 × 10^5^ tumor cells in 100 μl PBS, 0.2 ml/mouse). The day after B16F10 injection, every morning in the animal room, mice were orally administered, to ensure that each animal receive the exact dosage corresponding to each animal’s body weight, with DTIC or TH everyday for 20 days. Food and water were given ad libitum during the experimental period. The day after the last oral administration, the mice were sacrificed using the widely accepted method which is cervical spine dislocation and the lungs were collected, washed, the number of lung tumor colonies was counted, then frozen in liquid nitrogen and stored at − 80 °C for protein and RNA extractions. Mice body weight was recorded daily for 21 days.

### Western blotting

Total protein was extracted from B16F10 cells or mice lungs using Radio-Immunoprecipitation Assay (RIPA) buffer following the manufacturer’s instructions. SDS-PAGE (10%) was then carried out to resolve 10 μg of extracted protein sample, which was then transferred to a PVDF membrane (Merck Millipore, USA). Membranes were incubated with primary antibodies against MMP2, MMP9, and CD44 (obtained from Abcam, Cambridge, UK), at 4 °C overnight, then washed and incubated with secondary antibody, IRDye 800CW Donkey anti-rabbit IgG (LI-COR, Inc., NE, USA), at room temperature for 30 min. The signal was detected using the OdysseyFc Imaging System (LI-COR, Inc., NE, USA). All protein quantifications were normalized to β-actin expression level.

### Cell proliferation assay

Cell proliferation was assessed using the 3-(4,5-cimethylthiazol-2-yl)-2,5-diphenyl tetrazolium bromide or MTT (Dojindo, Japan) assay. B16F10 cells (3 × 10^4^ cells/well) were seeded onto 96-well plates and cultured as described above. After 24 h of incubation, the medium was replaced with medium without or containing TH or gnidilatidin at various concentrations. MTT (5 mg/ml) was then added, the plates covered with aluminum foil, and incubated further for 48 h. Sodium dodecyl sulphate (SDS; 10%) was then added at 100 μl per well, and incubated overnight at 37 °C to dissolve the formazan completely. Absorbances were obtained at 570 nm using a microplate reader (Powerscan HT; Dainippon Pharmaceuticals USA Corp., NJ, USA). Blanks containing medium only was used to correct the absorbances.

### Viable cell count

Viable cell count was performed using the ViaCount program of Guava PCA (Millipore, Billerica, MA, USA). B16F10 cells were seeded onto 10-mm dish (5 × 10^4^ cells/ml) and incubated for 24 h at 37 °C, after which, medium was replaced with fresh medium without or containing TH or gnilatidin. After further incubation for 48 h, cells were collected and resuspended in 1 ml medium and stained with DNA-binding Guava ViaCount reagent (Millipore) and analyzed according to the manufacturer’s instructions.

### Wound healing assay

B16F10 cells were seeded onto 6-well plates (5 × 10^5^ cells/well) until they reach 80% confluence when a scratch was made on the cell monolayer using a sterile white pipette tip, and washed with PBS (−) to remove the detached cells. The growth medium was then replaced by fresh growth medium without or with 1000 *v*/v TH or gnidilatidin. The cells were photographed using Leica DFC290 HD camera (Beckman Coulter, CA, USA) before scratching and at 0 h, 12 h, and 24 h following sample treatment. The images of the cells before and after scratching in treated and untreated cells were then compared.

### Cell adhesion assay

B16F10 cells were seeded onto 10-cm petri dishes (3 × 10^5^ cells/ml) and incubated for 24 h at 37 °C. After overnight incubation, the medium was replaced with TH- or or gnidilatidin-containing medium. After further incubation (24 h) cells were trypsinized and resuspended in serum-free RPMI 1640 medium (2 × 10^5^ cells/ml). The cell suspensions (100 μl) were then seeded onto fibronectin coated-plate (BD). After incubation for 1 h at 37 °C, medium was removed and washed three times with PBS (−) to remove the unattached cells, after which, MTT assay was perfomed as described above.

### Invasion assay

Invasion assay was performed using the 24-well Corning BioCoat Matrigel Invasion Chambers containing Falcon cell culture inserts (8 μh pore size PET membrane with a thin layer of Matrigel basement membrane matrix (Corning, Bedford, MA, USA), following the manufacturer’s instructions. B16F10 cells (5 × 10^5^ cells/ml), in serum-free media, were seeded onto the Matrigel-coated chambers while 10% FBS was added to the lower chamber as chemoattractant. After 24 h incubation, the lower chamber was stained with DAPI (Vector Laboratories, Inc., Burlingame, CA, USA), and the number of cells that had invaded moved to the lower chamber was counted using Leica DMI-4000B fluorescence microscope. Three fields in each sample were counted and the mean values from three independent experiments were used.

### DNA microarray analysis

DNA microarray was performed using Affymetrix (Santa Clara, CA, USA) GeneChip Mouse Genome 430 2.0 Array following the manufacturer’s instructions. Total RNA was extracted from B16F10 cells (3 × 10^5^ cells/ml) and the quality was assessed using Agilent 2100 bioanalyzer (Agilent Technologies). Biotin-labeled aRNA was synthesized by in vitro transcription and the purified aRNA (10 μg) was fragmented using the GeneAtlas 3’ IVT Express Kit, and was hybridized to the gene chip for 16 h at 45 °C. The chips were washed and stained in the GeneAtlas Fluidics Station 400 (Affymetrix) and the resulting image scanned using the GeneAtlas Imaging Station (Affymetrix). Identification of the differentially expressed genes was performed using Affymetrix® Expression Console™ Software and Affymetrix® Transcriptome Analysis Console (TAC) 2.0 Software (Affymetrix).

### Quantitative real-time PCR

RNA (1 μg), extracted using Isogen reagent (Nippon Gene, Tokyo, Japan), was reverse transcribed using the SuperScript III reverse transcriptase kit (Invitrogen, Carlsbad, CA, USA). Primers specific to *Egr*, *Id2*, *Sytl2*, *Tyr*, *Trp1*, *Dct*, and *Mitf* were used for real-time PCR performed using 7500 Fast Real-time PCR system using TaqMan Universal PCR mix and TaqMan probes (Applied Biosystems, Foster City, CA, USA). All reactions were run in triplicate, and data were analyzed using the 2 ^-**Δ Δ** C^_T_ values method.

### Statistical analysis

Results were expressed as mean ± standard deviations (SD), and the statistical evaluation performed using Student’s t-test when two value sets were compared (Control vs treated cells). ANOVA was performed to assess the level of significance between groups’ number of nodules and weight. A *P* value of ≤0.05 was considered to be statistically significant.

## Results

### Daphnane diterpenes suppressed B16F10 cells lung colonization

**Fig. 1:**
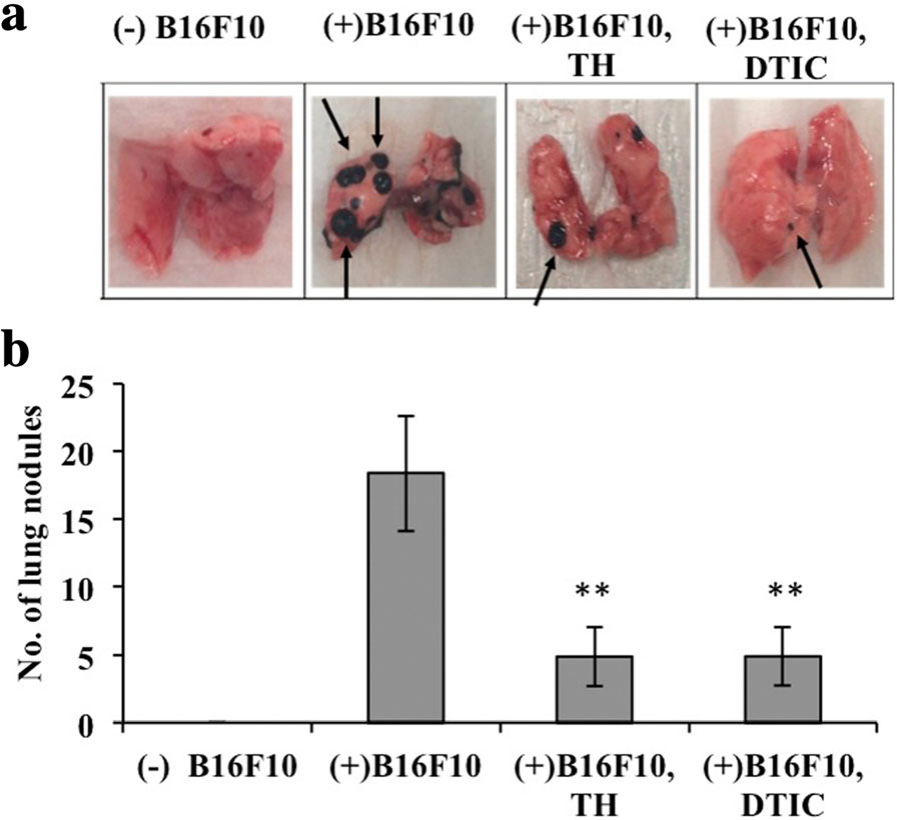
Effect of daphnane diterpenes on mice lung colonization by B16F10 cells. **a** Photographs of lungs of mice injected without (−)B16F10 or with (+) B16F10, and treated with dacarbazine (DTIC) or 1/1000 *v*/v *T. hirsuta* extract (TH). Arrows point to the lung nodules or tumors; **b** Plot of the number of mice lungs nodules (tumor) in mice injected without (−)B16F10 or with (+) B16F10, and treated with dacarbazine (DTIC) or TH. **Statistically significant (*P* ≤ 0.01) difference between treated cells and control

To investigate the effect of daphnane diterpenes on tumor cell adhesion to mice lungs, the lung tumors that formed from tail vein-injected B16F10 cells was counted after 3 weeks of oral administration with 50 mg/kg of daphnane diterpenes-rich *T. hirsuta* extract (TH) or 70 mg/kg/day dacarbazine (DTIC), the positive control. Compared to untreated mice (+B16F10), a reduction in tumor colonization (70%) was observed in “TH group” and “DTIC group” (Fig. 1a and b). The number of nodules was significantly different among groups (*P* < 0.00001). Tumors were observed in mice injected with B16 cells while none in mice injected with just water (Control). No significant difference in weight between mice groups was observed (*P* = 0.1) (Additional file 1: Table S1).

### Daphnane diterpenes decreased the metastasis-associated proteins expression in mice lungs

**Fig. 2:**
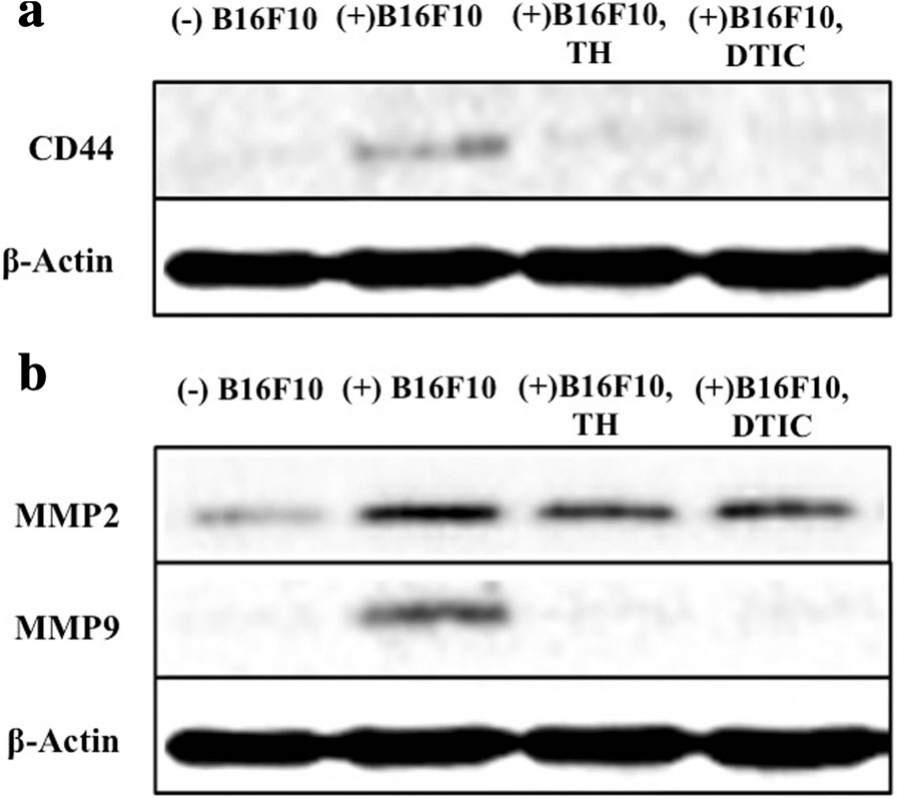
Effect of daphnane diterpenes on the expression of MMP2 and CD44 in vivo. The expression of (**a**) CD44 (**b**) and metalloproteinases MMP9 and MMP2 in mice injected without (−) B16F10 or with (+) B16F10, and treated with dacarbazine (DTIC) or 1/1000 v/v *T. hirsuta* extract (TH). Total proteins were then extracted and resolved by SDS-PAGE; the resolved proteins were then blotted onto a PVDF membrane. CD44, MMP2, and MMP9 were detected by immunoblotting with an anti-CD44, anti-MMP2, or anti-MMP9, polyclonal antibodies. The signals were visualized using LiCor Odyssey Infrared Imaging System after reaction with goat anti-mouse IRDye 680LT, or goat anti-rabbit IRDye 800CW (LI-COR). CD44, MMP2, and MMP9 levels were normalized to β-actin expression

CD44 and MMPs MMP2 and MMP9 are implicated in the progression and metastasis of melanoma [10]. Determination of the effect of daphnane diterpenes on CD44 expression showed that mice injected with B16 cells had higher CD44 level compared to the uninjected control mice. Treatment with either TH or DTIC significantly decreased CD44 expression (Fig. 2a,). In addition, B16 cells-injected mice had increased MMP2 and MMP9 levels (Fig. 2b) but after oral administration with TH or DTIC, MMP2 and MMP9 levels were significantly lowered (Fig. 2b, Additional file 2: Figure S1).

### Daphnane diterpenes have no effect on the proliferation and viability of B16F10 cells

**Fig. 3:**
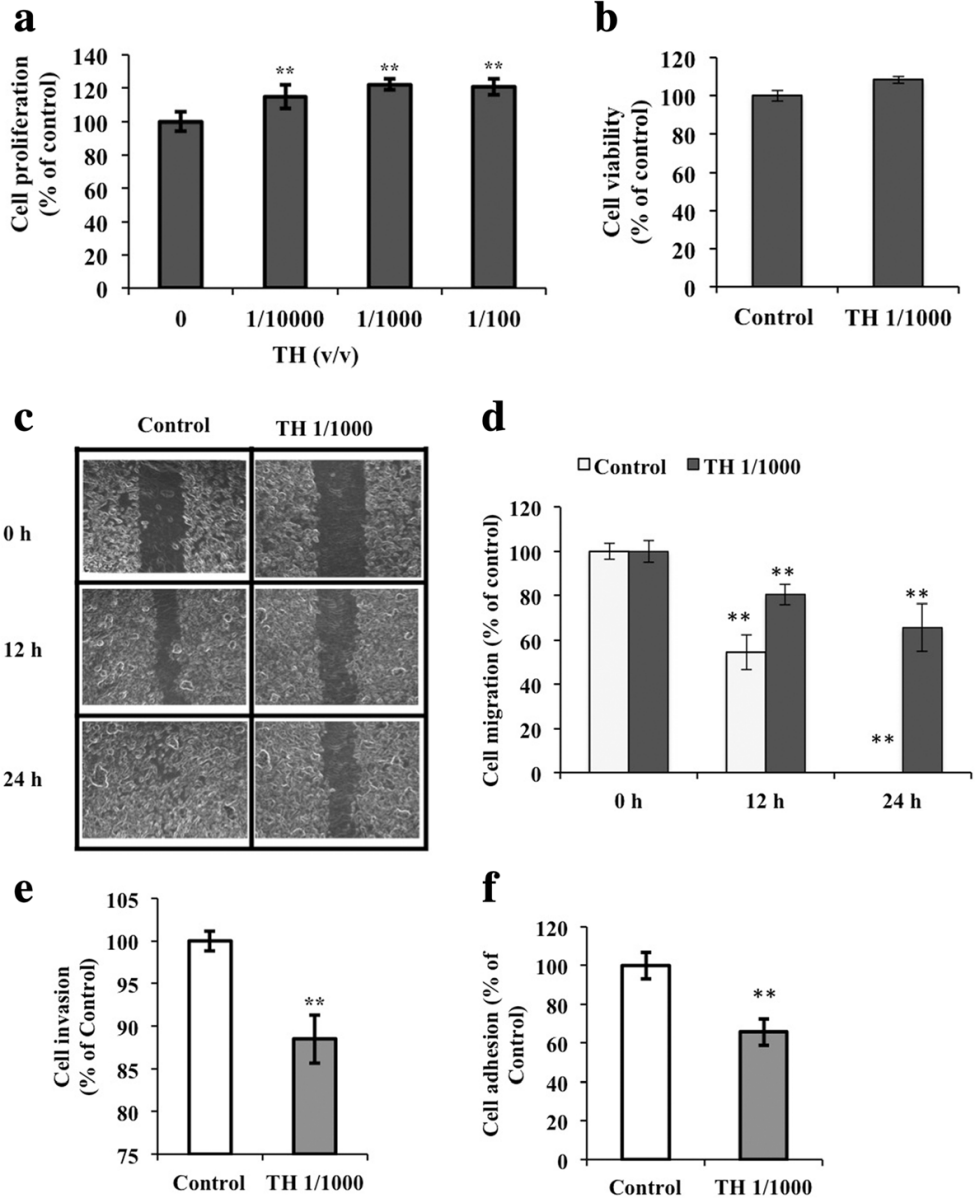
In vitro evaluation of the effect of daphnane diterpenes on B16F10 cells metastasis. **a** Cell proliferation evaluated using the MTT assay; **b** Viability of B16F10 cells treated with 1/000 v/v *T. hirsuta extract* (TH) evaluated using flow cytometry (Guava ViaCount assay); **c** Scratched cell monolayer images for the wound healing assay results (cell migration assay); **d** Wound healing assay results; **e** Cell invasion results obtained using the matrigel invasion assay; **f** Cell adhesion assay results evaluated by performing the MTT assay on B16 cells cultured in fibronectin-coated 96-well plates. Results represent the mean ± SD of triplicate samples. *Statistically significant (*P* ≤ 0.05) difference between treated cells and control. **Statistically significant (*P* ≤ 0.01) difference between treated cells and control

To establish if the observed decrease in MMPs and CD44 expression was not due to cytotoxicity of daphnane diterpenes, B16 cells proliferation and viability were evaluated using MTT assay and flow cytometry, respectively. Treatment with 1/10,000, 1/1000, or 1/100 TH for 24 h, 48 h, and 72 h had no significant change in the cell proliferation of B16 cells after 24 h at all concentrations tested (Fig. 3a). Extending the treatment time did not cause a significant change either except for 1/100 *v*/v TH-treated cells. Choosing 1000 v/v TH for the succeeding in vitro experiments, flow cytometry evaluation of cell viability showed that there was no significant difference between the control and TH-treated cells (Fig. 3b). No significant difference was observed in the number of control and TH-treated cells either (data not shown).

### Daphnane diterpenes inhibited cell migration, invasion, and cell adhesion

Results of wound healing analyses that monitored the cell movement between the gaps of the scratch made showed that compared to the control, TH decreased the gap created over a confluent monolayer after 12 h and 24 h, but not as fast as the control. In control (untreated cells) the gap completely closed after 24 h (Fig. 3c and d). Measurement of the ability of cells to degrade the basement membrane revealed that treatment with daphnane diterpenes (1/1000 *v*/v TH) inhibited the invasive ability of B16F10 cells by 12% (Fig. 3e). Evaluation of the B16F10 cells’ adhesion to the extracellular matrix (ECM) showed that adhesion on ECM-coated plates was significantly reduced (35%) by TH (Fig. 3f).

### Effect of daphnane diterpenoid gnidilatidin on global gene expression in B16F10 cells

**Fig. 4:**
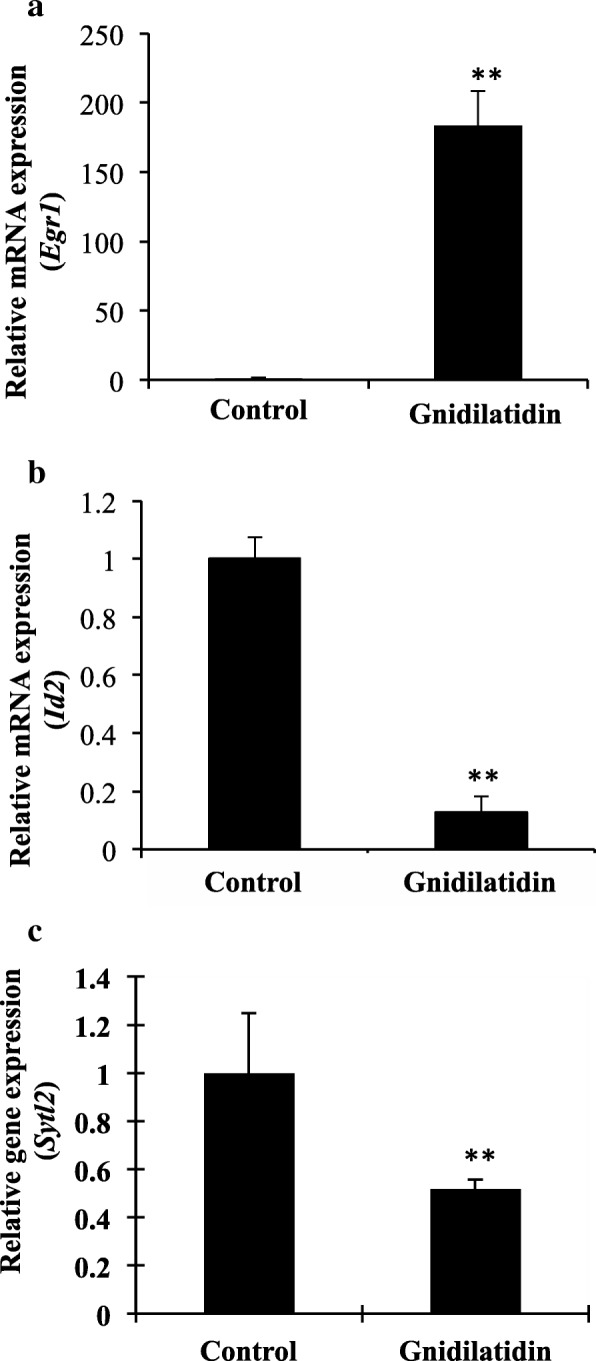
Quantitative real-time PCR of cancer metastasis-associated genes expression in 0.1 μM gnidilatidin-treated B16F10 melanoma cell lines. Relative gene expression levels of (**a**) Early growth response 1 (*Egr1*), (**b**) Inhibitor of DNA binding (*Id2*), and (**c**) Synaptogamin-like 2 (*Sytl2*) genes. Results represent the mean ± SD of triplicate samples. *Statistically significant (*P* ≤ 0.05) difference between treated cells and control. **Statistically significant (*P* ≤ 0.01) difference between treated cells and control

To examine the mechanism of the effect of daphnane diterpenes on melanoma, the global gene expression in B16F10 cells treated with gnidilatidin, a daphnane diterpene present in TH, was performed. Out of 39,000 transcripts, gnidilatidin upregulated 396 genes (> 2 expression ratio) significant for signal transduction (16%), apoptosis (32%), cell adhesion (19%), and cell cycle (33%), and downregulated 212 genes (< 0.5 expression ratio). The top 10 genes upregulated by gnidilatidin are *Egr1*, *Zfp36*, *Fos*, *Junb*, *Nr4a1*, *Ier2*, *Sgk*, *Cyr61*, *Vasn*, and *Sertad* while the downregulated genes are *Id2*, *Sytl2*, *Tbca*, *Sorbs1*, *Zfp85-rs1*, *Esco2*, *Mtss*, *Zfp60*, *Psg28*, and *Brca1* (Tables 1 and 2). The changes in the expression of *Egr1*, *Vasn*, *Id2*, and *Sytl2,* determined by microarray to be significantly up- or downregulated, were validated using quantitative real-time PCR. Results show that indeed, treatment with gnidilatidin for 1 h increased the expression level of the *Egr1* but decreased *Id2* and *Sytl2* expression (Fig. 4a-c).

**Table 1:** List of top ten (10) upregulated genes in gnidilatidin-treated B16F10 murine melanoma cells as determined by DNA microarray (Expression ratio of gnidilatidin vs Control)^a^

Gene	Function	Gnidilatidin (vs Control)
*Egr1,* early growth response 1	Required for differentiation and mitogenesis; a cancer suppressor gene; activates expression of p53/TP53 and TGFB1, and thereby helps prevent tumor formation.	113.24
*Zfp36,* zinc finger protein 36	Plays a role in the regulation of keratinocyte proliferation, differentiation and apoptosis; Plays a role as a tumor suppressor by inhibiting cell proliferation in breast cancer cells.	30.69
*Fos,* FBJ osteosarcoma oncogene *Junb,* Jun-B oncogene	FOS proteins have been implicated as regulators of cell proliferation, differentiation, and transformation; Transcription factor involved in regulating gene activity following the primary growth factor response	20.57
*Nr4a1,* nuclear receptor subfamily 4, group A, member 1	The encoded protein acts as a nuclear transcription factor. Translocation of the protein from the nucleus to mitochondria induces apoptosis; May inhibit NF-kappa-B transactivation of IL2. Participates in energy homeostasis by sequestrating the kinase STK11 in the nucleus, thereby attenuating cytoplasmic AMPK activation.	20.15
*Ier2,* immediate early response 2	DNA-binding protein that seems to act as a transcription factor; Involved in the regulation of neuronal differentiation, acts upon JNK-signaling pathway activation and plays a role in neurite outgrowth in hippocampal cells.	16.86
*Sgk1,* serum/glucocorticoid regulated kinase 1	Encodes a serine/threonine protein kinase that plays an important role in cellular stress response. This kinase activates certain potassium, sodium, and chloride channels, suggesting an involvement in the regulation of processes such as cell survival, neuronal excitability, and renal sodium excretion. Phosphorylates BRAF and MAP3K3/MEKK3 and inhibits their activity.	13.23
*Cyr61,* cystein rich protein 61	Promotes cell proliferation, chemotaxis, angiogenesis and cell adhesion. Appears to play a role in wound healing by up-regulating, in skin fibroblasts, the expression of a number of genes involved in angiogenesis, inflammation and matrix remodeling including VEGA-A, VEGA-C, MMP1, MMP3, TIMP1, uPA, PAI-1 and integrins alpha-3 and alpha-5. CYR61-mediated gene regulation is dependent on heparin-binding.	12.13
*Vasn,* vasorin	May act as an inhibitor of TGF-beta signaling.	11.03
*Sertad1,* SERTA domain containing 1	Stimulates E2F1/TFDP1 transcriptional activity. Renders the activity of cyclin D1/CDK4 resistant to the inhibitory effects of CDKN2A/p16INK4A.	10.33

^a^Data based on the average of three samples

**Table 2:** List of top ten (10) downregulated genes in gnidilatidin-treated B16F10 murine melanoma cells as determined by DNA microarray (Expression ratio of gnidilatidin vs Control)^a^

Gene	Function	Gnidilatidin (vs. Control)
*Id2,* inhibitor of DNA binding 2	Promotes tumor cell migration and invasion; The protein ID2 belongs to the inhibitor of DNA binding family, members of which are transcriptional regulators that contain a helix-loophelix (HLH) domain but not a basic domain.	0.16
*Sytl2,* synaptogaminlike 2	The SLP homology domain (SHD) of this protein has been shown to specifically bind the GTPbound form of Ras-related protein Rab-27A (RAB27A). This protein plays a role in RAB27Adependent vesicle trafficking and controls melanosome distribution in the cell periphery.	0.17
*Tbca,* tubulin cofactor a	Tubulin-folding protein; involved in the early step of the tubulin folding pathway.; The product of this gene is one of four proteins (cofactors A, D, E, and C) involved in the pathway leading to correctly folded beta-tubulin from folding intermediates.	0.19
*Sorbs1,* Sorbin and SH3 domaincontaining 1	This gene encodes a CBL-associated protein which functions in the signaling and stimulation of insulin. Mutations in this gene may be associated with human disorders of insulin resistance. Required for insulin-stimulated glucose transport. Involved in formation of actin stress fibers and focal adhesions (By similarity).	0.21
*Zfp85-rs1,* Zinc finger protein 85, related sequence 1	Regulation of transcription, DNA-templated; nucleic acid binding; metal ion binding; transcription corepressor activity	0.21
*Esco2,* Establishment Of Sister Chromatid Cohesion NAcetyltransferase 2establishment of cohesion 1 homolog 2	Acetyltransferase required for the establishment of sister chromatid cohesion. Couples the processes of cohesion and DNA eplication to ensure that only sister chromatids become paired together. In contrast to the structural cohesins, the deposition and establishment factors are required only during the S phase. Acetylates the cohesin component SMC3.	0.22
*Mtss1,* metastasis suppressor 1	May be related to cancer progression or tumor metastasis in a variety of organ sites, most likely through an interaction with the actin cytoskeleton.	023
*Zfp60,* zinc finger protein 60	Negative regulator of cartilage differentiation	0.25
*Psg28,* pregnancyspecific glycoprotein 28	Relevant for cell adhesion and for positive regulation of endocytosis, phagocytosis, and inflammatory response	0.26
*Brca1,* breast cancer 1	E3 ubiquitin-protein ligase that specifically mediates the formation of Lys-6-linked polyubiquitin chains and plays a central role in DNA repair by facilitating cellular responses to DNA damage. Required for appropriate cell cycle arrests after ionizing irradiation in both the Sphase and the G2 phase of the cell cycle. Involved in transcriptional regulation of P21 in response to DNA damage.	0.27

^a^Data based on the average of three samples

### Gnidilatidin inhibited B16F10 cells migration and adhesion

**Fig. 5:**
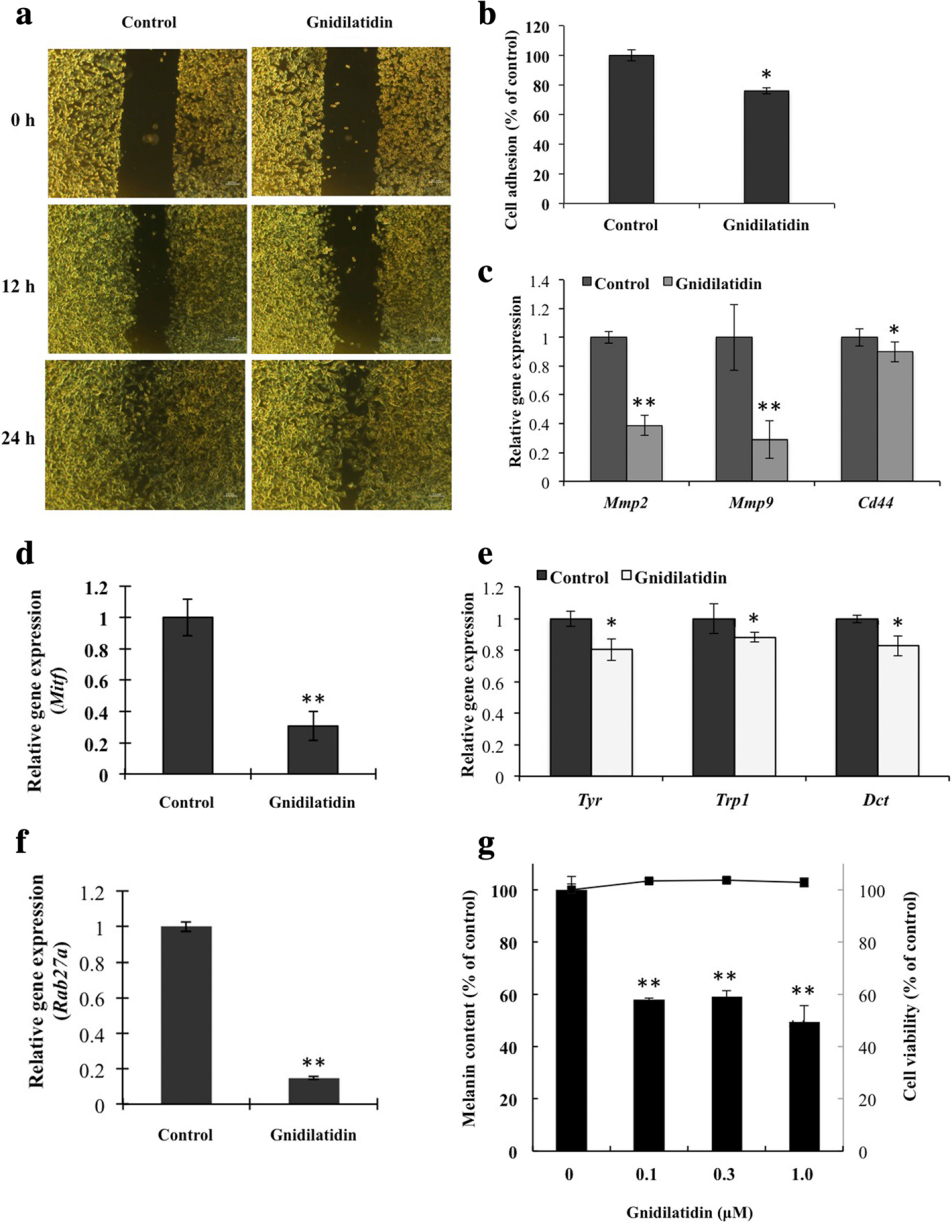
Effect of gnidilatidin (0.1 μM) on wound healing, cell adhesion, and *Mitf* expression in B16F10 melanoma cell lines. **a** Scratched cell monolayer images for the wound healing assay results (wound healing assay results; **b** Cell adhesion assay results evaluated by performing the MTT assay on B16 cells cultured in fibronectin-coated 96-well plates; **c** Relative gene expression of *Mmp2*, *Mmp9*, and *Cd44* genes; **d** Relative gene expression of *Mitf*; **e** Relative gene expression of tyrosinase (*Tyr*), **c** tyrosinase-related protein 1 (*Trp1*), dopachrome tautomerase (*Dct*), and (**f**) Ras-related protein (*Rab27a*), determined using TaqMan real-time quantitative PCR; **g** Melanin content of B16 cells. Results represent the mean ± SD of triplicate samples. *Statistically significant (*P* ≤ 0.05) difference between treated cells and control. **Statistically significant (*P* ≤ 0.01) difference between treated cells and control

Wound healing test on B16F10 cells treated with or without gnidilatidin showed a significant decrease in the ability of the Gnidilatidin-treated cells to “heal” the scratch made on the confluent cell layer (Fig. 5a). The same effect on wound healing was observed in SK-MEL-28 cells (data not shown). Evaluation of the B16F10 cells’ adhesion to the extracellular matrix (ECM) showed that adhesion on ECM-coated plates was significantly reduced (24%) by 24 h of gnidilatidin treatment (Fig. 5b). Determining the expression of extracellular membrane proteins’ genes expression showed a significant decrease in the expression of *Mmp2*, *Mmp9*, and *Cd44* genes.

### Gnidilatidin downregulated *Mitf* expression

MITF is the master regulator of melanocyte development, function, and survival differentiation but at the same time, promotes malignant behavior [20]. The effect of gnidilatidin on the melanin content, the expression of *Mitf* and to confirm the decrease in Mitf expression, the expression of *Mitf*-regulated genes important for melanogenesis such as tyrosinase (*Tyr*), tyrosinase-related protein 1 (*Trp1*), and dopachrome tautomerase (*Dct*), as well as the melanosome transport protein *Rab27a* was then determined. Gnidilatidin downregulated *Mitf* expression by 30% after 4 h of treatment, causing a decrease in the expression of the *Tyr*, *Trp1*, *Dct*, and *Rab27a* (Fig. 5c-e). Moreover, gnidilatidin-decreased expression of the melanogenic enzymes (to 85%) caused a dose-dependent melanogenesis inhibition (50%) (Fig. 5d).

## Discussion

The use of natural products to prevent cancer has been recognized to be effective. Tea catechins, for example, have been demonstrated to have significant anti-cancer and cancer metastasis preventive effects [21]. Moreover, the use of natural products against melanoma and melanoma metastasis, in particular, has been the focus of several studies recently [22, 23]. In this study, we demonstrated the potential of daphnane diterpenes as therapeutics against melanoma metastasis. Based on the in vivo tumorigenesis assay, treatment with daphnane diterpenes-rich *T. hirsuta* extract (TH) decreased the number of lung tumors in B16-injected mice (Fig. 1a-b) without affecting mice weight and without cytotoxicity (Additional file 1: Table S1, Fig. 3a) and comparable to treatment with DTIC. The metastatic process requires the following steps: attachment of the cells to the matrix components, local degradation of the matrix by metalloproteinases, and migration of the cells to other parts of the body. Daphnane diterpenes inhibited not just cell migration, but also cell invasion and adhesion (Fig. 3c-f).

The recruitment of cell adhesion molecule CD44 on the surface of the cells is an important metastasis event. CD44 regulates progression and metastasis of several cancers such as breast cancer, prostate cancer, and melanoma [10, 24–27] by interacting with extracellular matrix that promotes cell motility [7] and has an affinity for other ligands, including matrix metalloproteinases (MMPs). MMPs are proteolytic enzymes and by degrading proteins, regulate the tumor environment, including cell growth, differentiation, and angiogenesis. MMPs expression and activation is therefore increased in almost all human cancers, including melanoma [28, 29]. Studies have shown that CD44 functions as a docking molecule for MMP9, suggesting a mechanism for CD44 cell migration [10]. Treatment with daphnane diterpenes-rich TH suppressed both CD44 and MMPs (MMP2 and MMP9) expression in vivo (Fig. 2a-b) preventing the formation of a CD44/MMP9 complex on the cell surface is indispensable for MMP9 activity [10].

Targeting MMPs for melanoma is also recognized as a promising therapeutic strategy. Synthetic MMP inhibitors have been looked to prevent metastasis. And while some reached clinical trials, some were prematurely terminated due to either lack of benefits or they cause unwanted effects [28]. The metastasis inhibitory effects of daphnane diterpenes without cytotoxicity (Fig. 3a and b) can be attributed to the six daphnane diterpenoids contained in TH [18, 19]. Further evaluation of the effect of daphnane diterpenes on B16F10 cells using TH daphnane diterpenoid gnidilatidin revealed the mechanism involved in its effect on metastasis. Gnidilatidin has a fatty acid-containing structure and is an analogue of the anticancer compound mezerein. Gnidilatidin (0.1 μM) has inhibitory effect on metastasis-associated genes and was not cytotoxic at up to 1.6 μM. Highly upregulated gene, immediate early gene (*Egr1*) (Table 1), encodes a nuclear, zinc finger protein and its transcription directly regulates multiple tumor suppressors such as TGFbeta, PTEN, p53, and fibronectin, as well as the *Sgk1* gene that phosphorylates BRAF and MAP3K3 and inhibit their activities [30]. Furthermore, induction of *Egr1* expression is associated with a decreased metastatic and invasive ability of human hepatocarcinoma cells [31]. Downregulated gene (Table 2) *Id2* is relevant in the inhibition of helix-loop-helix transcription factors such as MITF [32] while *Sytl2* binds the GTP-bound form of Ras-related protein Rab27a [33]. Id proteins (e.g. Id2) have been reported to interact with MITF and inhibit its activity [32]. In vitro analysis of the effect of daphnane diterpene gnidilatidin on B16F10 cell migration and adhesion showed that it appeared to have slowed down the healing of the gap in scratched cell monolayer in the wound healing assay (Fig. 5a) and decreased cell adhesion (Fig. 5b) and this can be attributed to the downregulation of the *Mmp2*, *Mmp9*, and *Cd44* gene expression (Fig. 5c).

We have previously reported that daphnane diterpenes can downregulate *Mitf* [34]. Here, it is most likely that gnidilatidin-induced inhibition of *Mitf* (Fig. 5d) was caused by the downregulation of Id2, causing a decrease in *Rab27a* and melanogenic enzymes’ genes *Tyr*, *Tyrp1*, and *Dct* (Fig. 5e) expression, and this effect was further validated by the inhibition of melanin content of the cells presented in Fig. 5g. Enhanced RAB27A expression has been reported to promote not only melanosome transport, for effective pigmentation, but also invasiveness and metastasis in breast cancer cells [35] (Fig. 5f). Rab27a has also been shown to be involved in exocytosis of insulin and chromaffin granules in endocrine cells. Autocrine and paracrine cytokines are essential for invasion and metastasis in some solid tumors and that its inhibition may also be another effective strategy to prevent tumor metastasis. Moreover, as overexpression of Rab27a protein is relevant in the redistribution of the cell cycle, daphnane diterpenes -rich TH- and gnidilatidin-induced downregulation of *Rab27*, therefore, lowers the cancer cells invasiveness and metastatic abilities.

MITF indirectly regulates CD44 and MMP2 through the *MET* proto-oncogene by binding and activating the *MET* promoter [36]. Reduction of MITF activity sensitizes melanoma cells to chemotherapeutic agents and targeting MITF has been suggested to be a rational therapeutic avenue into highly chemotherapy-resistant melanoma [15]. Whether the decreased level of CD44 and MMP9 following daphnane diterpenes-rich TH or gnidilatidin treatment (Fig. 2a and b) was a result of *Mitf* inhibition was not determined but is most likely.

Since TH and its daphnane diterpenes components have antimelanogenesis effect [13, 14], it is not surprising that gnidilatidin has the same effect (Fig. 5e). The presence of other daphnane diterpenoids in TH at the right concentration, and possibly in synergy with each other, gave it its beneficial effects against metastasis and melanoma. It will be interesting to find out the effect of TH on the inflammatory system in vivo, as well as its effect on other types of cancer. Clinical trials using TH would be interesting and would enable us to explore its possible cancer-preventive effects.

## Conclusions

Our results demonstrated for the first time that treatment with daphnane diterpenes can significantly inhibit lung metastasis of B16F10 cells, as demonstrated by the inhibition of B16F10 cells migration, invasion, and adhesion, associated with suppression of CD44 and MMPs (MMP2 and MMP9) expression. Moreover, treatment with gnidilatidin has shown that at the signal transduction level, gnidilatidin increased the expression of MITF and the direct regulator of tumor suppressors, *Egr1*, accompanied by a downregulation of tumor cell migration and invasion-associated gene *Id2*, providing further proof on the potential of daphnane diterpenes for use as therapeutics against melanoma metastasis, either alone or in combination with other daphnane diterpenes or existing anti-cancer drugs.

## Additional files

Additional file 1: **Table S1:** Weekly weight measurements of all mice groups from day 1 to 21. It is a table showing the body weight of the animals. (PDF 130 kb)

Additional file 2: **Figure S1:** The protein bands intensity of MMP2 and MMP9 obtained using Li-COR Software. (PPT 191 kb)

## Abbreviations

B16F10: B16 murine melanoma cells
CD44: CD44 antigen
DTIC: Dacarbazine
MITF: Microphthalmia-associated transcription factor
MMP2: Matrix metallopeptidase 2
MMP9: Matrix metallopeptidase 9
MTT: 3-(4,5-cimethylthiazol-2-yl)-2,5-diphenyl tetrazolium bromide
TH: *Thymelaea hirsuta* extract
