# Correction to: Daphnane diterpenes inhibit the metastatic potential of B16F10 murine melanoma cells in vitro and in vivo

**DOI:** 10.1186/s12885-018-4805-8

**Published:** 2018-09-18

**Authors:** Myra O. Villareal, Yuki Sato, Kyoko Matsuyama, Hiroko Isoda

**Affiliations:** 1 Faculty of Life and Environmental Sciences, University of Tsukuba, Tsukuba City, 305–8572 Japan; 2 Alliance for Research on the Mediterranean and North Africa (ARENA), University of Tsukuba, Tsukuba City, 305–8572 Japan; 3 Graduate School of Life and Environmental Sciences, University of Tsukuba, 305, Tsukuba City, –8572 Japan

## Correction to: BMC Cancer (2018) 18:856 10.1186/s12885-018-4693-y

Following publication of the original article [1], the authors reported that affiliation number 2 was incomplete. In this Correction, correct and incorrect affiliations are shown.

Incorrect affiliation:

Alliance for Research on North Africa (ARENA), University of Tsukuba, Tsukuba City 305–8572, Japan.

Correct affiliation:

Alliance for Research on the Mediterranean and North Africa (ARENA), University of Tsukuba, Tsukuba City 305–8572, Japan.

In addition, caption “*Statistically significant (*P* ≤ 0.05) difference between treated cells and control” was deleted from Figs. 3 and 4 in the original article.
