# Supplementary material for: Daphnane diterpenes inhibit the metastatic potential of B16F10 murine melanoma cells in vitro and in vivo
Source: BMC Cancer. 2018 Aug 29;18:856. doi: 10.1186/s12885-018-4693-y (PMC6116488; doi:10.1186/s12885-018-4693-y)
Supplement: Supplementary file 1 — Table S1: Weekly weight measurements of all mice groups from day 1 to 21. It is a table showing the body weight of the animals. (PDF 130 kb) [file 12885_2018_4693_MOESM1_ESM.pdf]

Table S1. Weekly weight measurements of all mice groups from day 1 to 21.

Treatment	Day 1	Day 7	Day 14	Day 21
(-) B16F10	21.0 ± 1.00	21.7 ± 1.16	22.2 ± 0.96	22.7 ± 0.86
+ B16F10	21.0 ± 0.79	21.4 ± 0.74	22.1 ± 0.82	21.3 ± 1.68
+ B16F10/+ TH	20.9 ± 0.48	21.4 ± 0.74	22.1 ± 0.69	21.3 ± 1.91
+B16F10/+ DTIC	21.1 ± 0.90	21.7 ± 1.10	22.1 ± 0.86	22.1 ± 1.06
