# Supplementary figures and images for: Daphnane diterpenes inhibit the metastatic potential of B16F10 murine melanoma cells in vitro and in vivo
Source: BMC Cancer. 2018 Aug 29;18:856. doi: 10.1186/s12885-018-4693-y (PMC6116488; doi:10.1186/s12885-018-4693-y)

## Slide 1
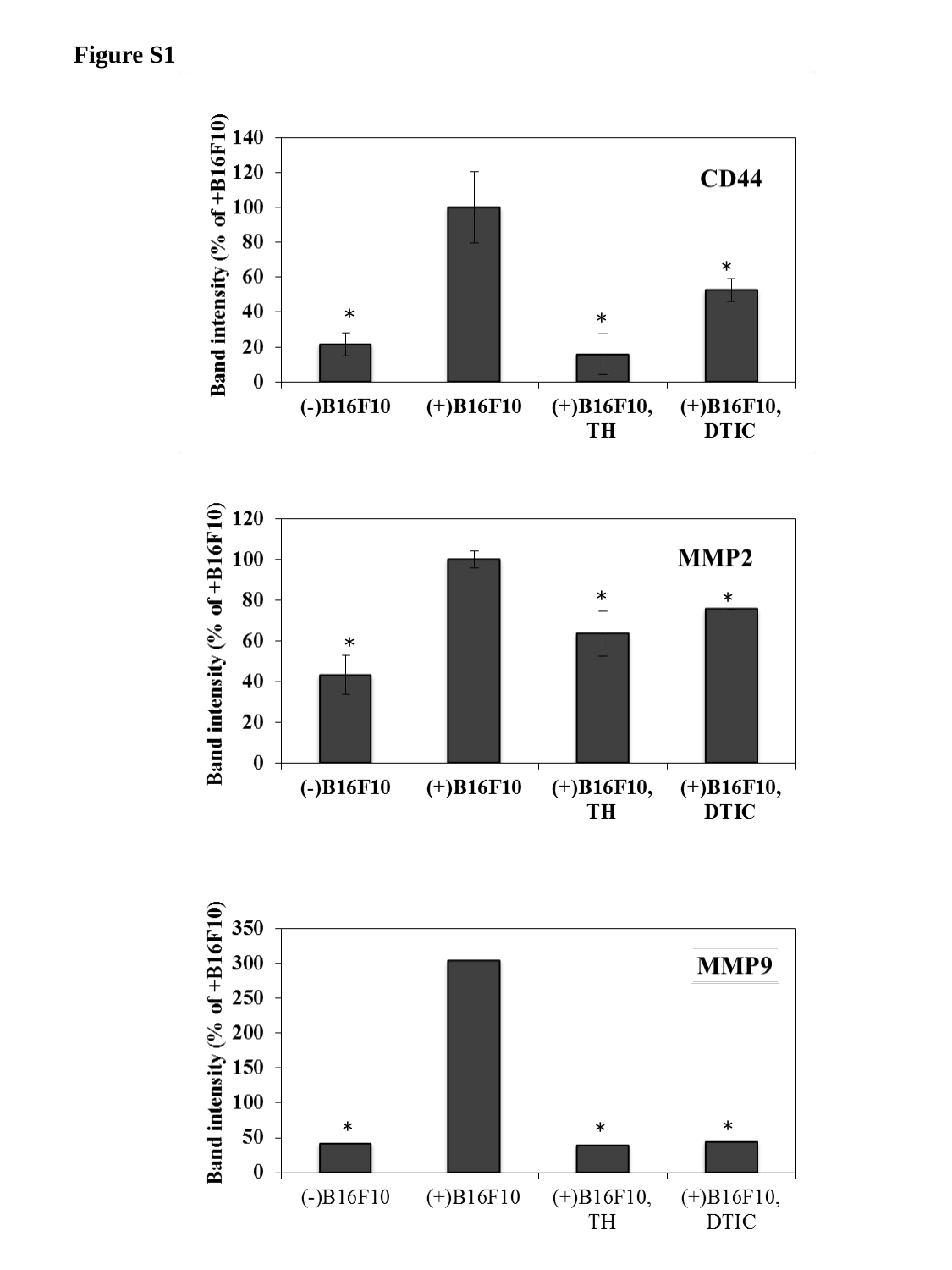

Figure S1
*
*
*
*
*
*
*
*
*

Supplement: Supplementary file 2 — Figure S1: The protein bands intensity of MMP2 and MMP9 obtained using Li-COR Software. (PPT 191 kb) [file 12885_2018_4693_MOESM2_ESM.ppt]
